# Reliability of automated topographic measurements for spine deformity

**DOI:** 10.1007/s43390-022-00505-9

**Published:** 2022-05-08

**Authors:** Benjamin N. Groisser, Howard J. Hillstrom, Ankush Thakur, Kyle W. Morse, Matthew Cunningham, M. Timothy Hresko, Ron Kimmel, Alon Wolf, Roger F. Widmann

**Affiliations:** 1 Department of Mechanical Engineering, Technion-Israel Institute of Technology, Haifa, Israel; 2 Department of Rehabilitation, Hospital for Special Surgery, New York City, NY USA; 3 Department of Orthopedic Surgery, Hospital for Special Surgery, New York City, NY USA; 4 Department of Orthopedic Surgery, Boston Children’s Hospital, Boston, MA USA; 5 Department of Computer Science, Technion-Israel Institute of Technology, Haifa, Israel

**Keywords:** Scoliosis, Reliability, Surface topography, Trunk shape, ATR

## Abstract

**Purpose:**

This study introduces a novel surface-topographic scanning system capable of automatically generating a suite of objective measurements to characterize torso shape. *Research Question*: what is the reliability of the proposed system for measurement of trunk alignment parameters in patients with adolescent idiopathic scoliosis (AIS) and controls?

**Methods:**

Forty-six adolescents (26 with AIS and 20 controls) were recruited for a prospective reliability study. A series of angular, volumetric, and area measures were computed from topographic scans in each of three clinically relevant poses using a fully automated processing pipeline. Intraclass correlation coefficients (ICC(2,1)) were computed within (intra-) and between (inter-) raters. Measurements were also performed on a torso phantom.

**Results:**

Topographic measurements computed on a phantom were highly accurate (mean RMS error 1.7%) compared with CT. For human subjects, intra- and inter-rater reliability were both high (average ICC > 0.90) with intrinsic (pose-independent) measurements having near-perfect reliability (average ICC > 0.98).

**Conclusion:**

The proposed system is a suitable tool for topographic analysis of AIS; topographic measurements offer an objective description of torso shape that may complement other imaging modalities. Further research is needed to compare topographic findings with gold standard imaging of spinal alignment, e.g., standing radiography. *Conclusion:* clinical parameters can be reliably measured in a fully automated system, paving the way for objective analysis of symmetry, body shape pre/post-surgery, and tracking of pathology without ionizing radiation.

**Supplementary Information:**

The online version contains supplementary material available at 10.1007/s43390-022-00505-9.

## Introduction

### Background

Idiopathic scoliosis is a complex 3-dimensional spinal deformity defined as a lateral curve in the frontal plane of 10 degrees or more associated with vertebral rotation. While clinicians tend to focus on curve magnitude and progression, patients and families are often concerned with thoracic prominence as well as shoulder, trunk, and waist-crease asymmetry [1–3]. Validated assessment tools and classification systems for scoliosis have been developed based on geometric radiographic measurements [4–6], but only recently have surface-topographic measures been recognized as important, objective measurements that may correlate closely with both patient self-image and radiographic measures of deformity [7–9].

The gold standard imaging modality for diagnosis and assessment of scoliosis remains radiography. The typical braced patient may receive 16 spine radiographs throughout their course of treatment [10], and despite widespread adoption of low-dose imaging systems [11], scoliosis patients experience elevated risk of carcinogenesis [12]. Furthermore, radiographic measurements correlate poorly with patient-reported outcomes measures (PROMs) especially in relation to self-image and appearance [13–15].

Topographic scanning has become an integral tool for surgical planning and assessment in surgical subspecialties including craniofacial reconstruction [16, 17] and breast surgery [18] where symmetry is a primary objective. Widespread adoption by orthopedic surgeons has been hampered by (1) lack of reimbursement codes, (2) scan time, (3) requirement for fiducial markers, (4) complexity and need for engineering expertise, (5) variable reliability, and (6) lack of standardized topographic measures. As a result, the use of surface-topographic scanning for scoliotic assessment has largely been confined to research or academic settings.

Despite these hurdles, many experimental and commercial systems have attempted to measure scoliotic trunk shape using moiré topography [19], structured light [20, 21], and laser scanners [22]. Notably, the Formetric 4D video-rasterstereography system is commercially available, and demonstrates good-to-excellent intra- and inter-rater reliability (most ICCs > 0.7) for several surface-topographic measurements in Adolescent Idiopathic Scoliosis (AIS) patients and allows for comparisons over time [23–25]. Crucially, for patients’ self-image, a recent experimental version of the scanner can capture 360° torso reconstructions [26]. However, measurements require manual landmarking by a trained technician and the system only operates in upright posture, precluding functional analysis (e.g., bending/twisting).

Inexpensive, accurate, reliable, and fast surface scanning techniques coupled with standardized surface-topographic measurements may pave the way for a larger role for topographic analysis in the diagnosis and treatment of scoliosis. Many prior studies have shown that topographic measurements can detect progression of scoliosis, thereby reducing the need for ionizing imaging in disease monitoring [25, 27, 28]. Beyond this, we believe that topographic measurements may ultimately surpass radiography in providing objective measures that correlate more closely with self-image. Topographic data can also be used to assess the impact of physical therapy, bracing, and surgery on objective surface measurements that are essential to evidence-based decision-making in orthopedic surgery.

### Contributions

Recent advances in computing power, coupled with affordable and accurate 3D scanners, set the stage for widespread proliferation of surface topography in many areas of medicine. Here, we describe a markerless surface scanning protocol coupled with a rapid, fully automated analysis pipeline to produce a suite of highly reliable surface-topographic measurements for patients with AIS. Any high-resolution surface scanner can be used, as the analysis software is agnostic to the underlying hardware. Designed for clinical practice, the system is:

1. Straightforward: no specialized training is needed to operate the system, and no fiducials/manual landmarks are required.
2. Automated: after data collection, no human intervention is required to produce topographic measurements.
3. Fast: each pose takes seconds to capture, while automated analysis takes several minutes.
4. High-fidelity: torso reconstructions have sub-millimeter accuracy and geometric measurements have near-perfect reliability.

To demonstrate the system’s utility, we perform the following **validation assessments**:

- 1. Surface reconstructions are accurate [Sect. 3.2].
- 2. Surface measurements on a rigid phantom are accurate [Sect. 3.3].
- 3. Surface measurements on human patients/controls are reliable [Sect. 3.4].

## Materials and methods

### Scanning hardware

All 3D scans were collected using the 3dMDbody system (3dMD, Atlanta, GA, USA). The photogrammetric scanner comprises 10 "Modular Camera Units" each of which includes two black-and-white stereo vision cameras and one RGB camera for a total of 30 cameras (details in Appendix A). The model in question features a capture rate of 10 frames per second with 1.8 ms exposure time and an operating volume of 1.2 × 2.2x2.2 m^3^.

### Subjects

Subjects were recruited from the Division of Pediatric Orthopedics at The Hospital for Special Surgery (New York City, NY, USA). The internal Institutional Review Board approved the Spinal Alignment Registry which comprises several analysis plans including this reliability study; informed assent and consent was obtained from subjects and their parents.

Inclusion criteria for patients were: 11 to 21 years of age and scheduled for whole-body biplanar radiographs for evaluation of spinal deformity. Patients with prior chest wall or spinal surgery, significant medical conditions or that were unable to stand independently were excluded. Control subjects of the same age were recruited from the Sports Medicine and Shoulder Service of pediatric orthopedics. Controls with a history of spinal deformity, asymmetry, prior chest wall or spinal surgery, significant medical conditions, or unable to stand independently were excluded.

All subjects underwent standard clinical examination and whole-body optical scans. Spinal deformity patients also had EOS biplanar radiographs as part of standard of care.

### Scan protocol

Subjects removed jewelry and glasses prior to changing into form-fitting clothing: low-waisted compression shorts and hairnets for all subjects, and custom halter tops exposing the back for females. The uniform is similar in price to a hospital gown and suitable for radiographs.

**Fig. 1:**
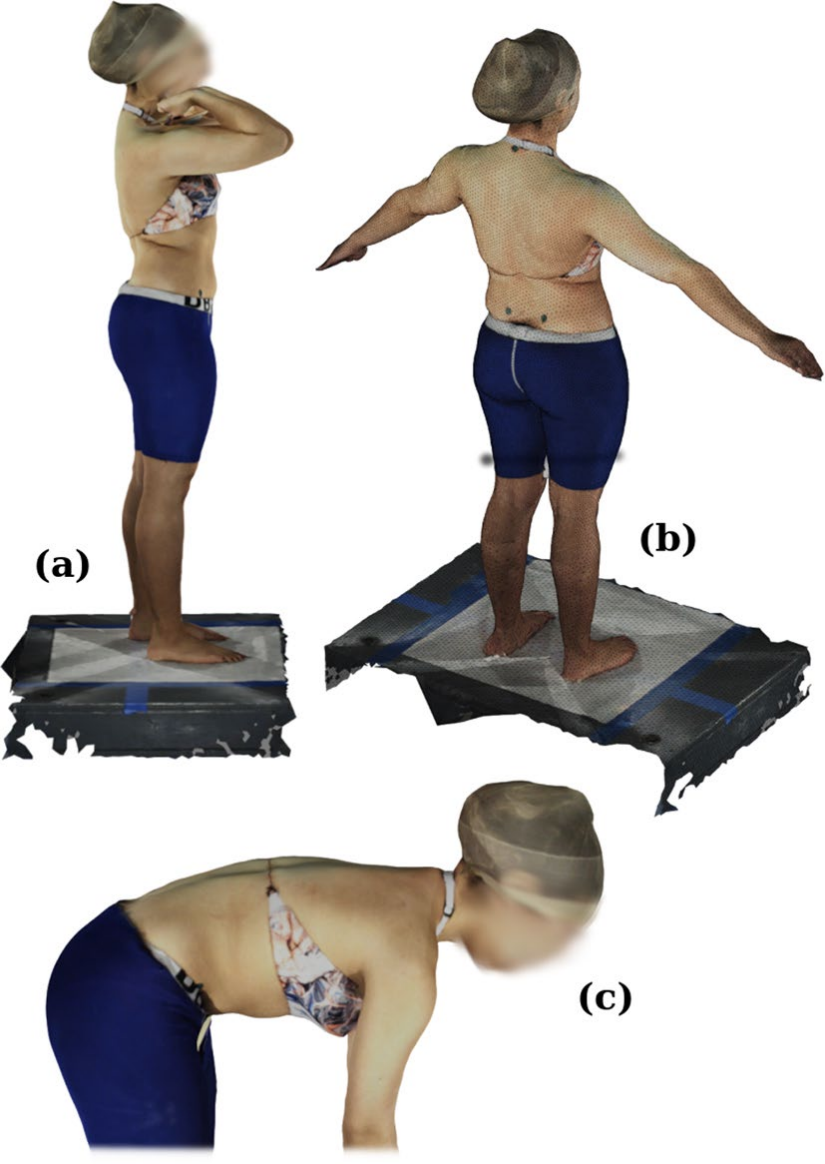
Surface scans are automatically reconstructed with RGB texture by the 3dMDbody system. **a** EOS pose, as used for biplanar radiographic scanning; **b** A-pose, a neutral standing posture; **c** Adam's forward bending posture. The A-pose reconstruction shows the mesh topology. Fiducial markers are used *only* to assess registration accuracy and not for any part of the automated processing pipeline

Subjects stood in the center of the optical scanner and were guided through a series of poses by a technician (Fig. 1). For this study,^1^ the following postures were selected as the most clinically relevant:

**EOS-pose** Feet were spaced at hip width with the right foot 2.5" anterior to the left. Elbows are bent with fingertips on shoulders.

**A-pose** Subjects marched in place before stopping in their natural angle and base of gait, fully erect with forward gaze and arms abducted 45°.

**Adam’s Bend** Feet were shoulder width apart, palms pressed together. Knees were fully extended, while the subject bent forward until the back was horizontal to the floor [29].

Each pose was scanned twice without change of foot position (test–retest). Participants then stepped out of the scan area before recording all sequences again (remove-replace). Finally, the entire process was performed with a second observer. The order of the two raters was randomized for each subject. Subjects were blind to the parameters being computed, while the analysis was fully automated and therefore insensitive to subject identity.

### Measurements

**Fig. 2:**
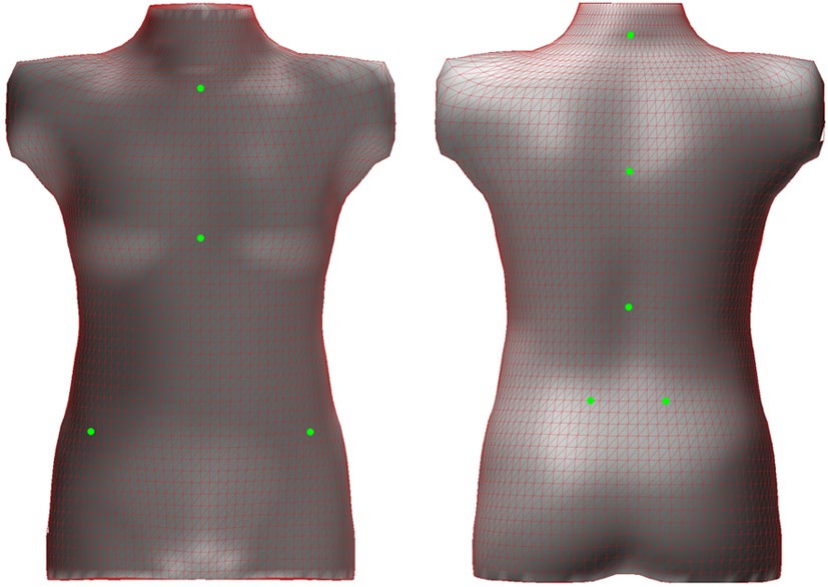
The torso template atlas has a symmetric grid connectivity pattern. Nine landmark locations are shown (described in the text), but an unlimited number of points, curves, areas, or volumes can be defined with reference to the template mesh and then applied to all registered scans

The input to our analysis software is a raw surface scan generated by the 3dMD scanner.^2^ A generic human torso “atlas” (Fig. 2) is deformed to fit the raw scan data. The output of this registration is a clean (topologically watertight manifold) torso and full anatomical correspondence with the atlas. For this study, we refer to nine relevant landmarks: Posterior Superior Iliac Spines (PSIS, bilateral), Anterior Superior Iliac Spines (ASIS, bilateral), the Xiphoid Process (XP), Jugular Notch (JN), and the spinous processes for L2, T8, and C7.

**Fig. 3:**
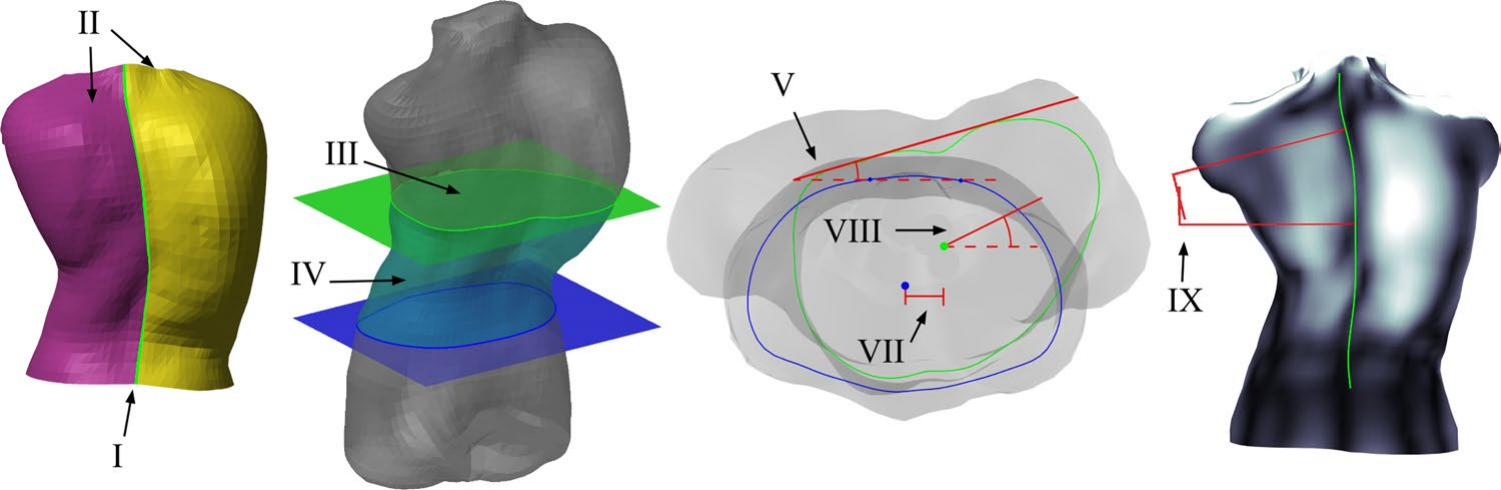
Surface topographic measurements; descriptions appear in the text with details in Appendix B. [I] Spine Length (green) is the midline arclength. [II] Back Area is the summation of left (magenta) and right (yellow) surface areas. [III] Cross-Section Area (green) is the area enclosed by the intersection of the torso surface and transverse plane. [IV] Cross-Section Volume is the volume of the portion of the torso bounded by transverse cuts (blue and green). [V] Back Surface Rotation (aka Angle of Trunk Rotation) measures the angle between a line tangent to the back and the coronal plane. [VII] Centroid Deviation is the lateral shift between the barycenter of an axial slice (green) compared to the centroid at the level of the PSIS (blue). [VIII] Axial Rotation is the angle between the principal axis of a transverse slice (green) and the coronal plane. [IX] Qangle is the topographic analogue of the Cobb angle using the dorsal symmetry line

From prior surface-topographic scanning literature, we selected nine specific measurements applicable to clinical spinal deformity practice (Fig. 3). We classified these measurements as either intrinsic (constant under rigid transformation) or pose-dependent (sensitive to orientation and/or minor postural change). For technical details of the registration and measurement algorithms, see Appendix B.

*Intrinsic measures*

I. **Spine length** Arclength of the midline from the PSIS centroid to C7.
II. **Back area** Surface area of the dorsal torso, bounded cranially by C7 and caudally by PSIS [30].
III. **Cross-sectional area **♱ Enclosed area of transverse plane slices taken through the torso at L2, T8, and JN [26]. Abbreviated XSA.
IV. **Section volume **♱ Three torso volumes were computed, bounded caudally/cranially with axial planes: (1) L2 to T8, (2) XP to JN, and (3) PSIS to JN [26]. Abbreviated XSV.

*Pose-dependent measures*

XXII. **ATR/BSR Max §** Trunk surface rotation is the angle of the line lying tangent to the back surface [31, 32] in reference to either (a) floor plane for Adam’s bend scans, or (b) the patient’s coronal plane for upright postures.
XXIII. **ATR/BSR X%** Trunk surface rotation was measured (as above) at predetermined intervals between PSIS and C7: 25%, 50%, and 75%.
XXIV. **Centroid deviation **♱** §** Centroid (barycenter of axial slice) deviation in the coronal plane, with reference to the PSIS slice centroid [33].
XXV. **Trunk axis **♱** §** Angle of the principal axis of transverse slices [33].
XXVI. **Qangle **♱ Analogous to Cobb angle, as in the Qantec [34] system; the back symmetry line [35] was fitted with a fourth-order harmonic function.

♱ Standing poses only

§ Maximum absolute value anywhere on the trunk reported

#### Statistical analysis

Intraclass correlation coefficients (ICC(2,1)) and standard deviations (SD) were computed for each parameter and pose using SPSS (version 25, IBM, Armonk, NY). All ICC calculations were made for absolute agreement, and lower/upper bounds were computed at the 95% confidence interval. Accuracy measures were reported as Root-Mean-Squared (RMS) error, also called the quadratic mean

$$RMS = \sqrt {\frac{1}{n}\mathop \sum \limits_{i}^{n} \left( {x_{i} } \right)^{2} .}$$

To assess the reliability of our methods for different body types, we compute Spearman correlation coefficients between subject Body Mass Index (BMI) and inter-rater topographic parameter consistency (relative difference for intrinsic measures and absolute difference for pose-dependent). A total of 36 parameters (from three poses) were tested, and then, the Bonferroni–Holm method was applied to correct for multiple comparisons (alpha = 0.05).

### Rigid-body scan targets

To validate the reconstruction accuracy of the scanner, a calibration target (aluminum optical breadboard) with evenly spaced fiducials was scanned in 3dMD. Planar reconstruction accuracy was evaluated by fitting a plane to the 3D surface and computing point-to-plane distances. For absolute error, fiducial landmarks were manually identified and distances between neighbors were measured.

**Fig. 4:**
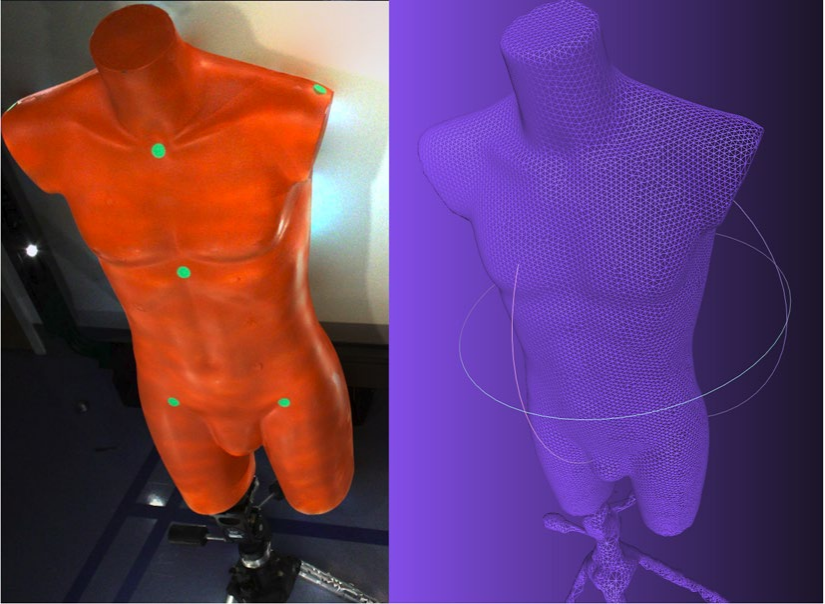
The phantom model is a rigid torso mannequin mounted to a tripod. The left image shows a cropped RGB image captured by 3dMD, while the right image shows the reconstructed mesh. Note that the fiducial markers are not used for any part of our analysis

To control for nonrigid postural variation, we simulated the scan protocol on a lightweight torso mannequin (Fig. 4). Ten repeated trials were performed in the upright position mounted to a tripod and the forward bend position lying prone on a small table. After fitting the torso template to the reconstructed scans, we computed the previously described measurements.

For comparison with a gold standard reference modality, we also scanned the torso mannequin with computed tomography (CT) at 970 × 970 × 625 μm voxel resolution (Discovery 750 HD, General Electric, Boston, USA). The surface was reconstructed using a marching cubes algorithm, and then fed into the automated measurement software for direct comparison with topographic scanning.

## Results

### Data collection and processing times

Demographic data of participants are shown in Table 1; Cobb angles were measured using EOS reconstructions [36]. On a five-patient sample, the total average time for optical scanning (one scan in each of three poses) was 2.7 min, while EOS imaging averaged 2.5 min per radiograph. Automated data processing takes approximately 10 min per subject including 3D reconstructions, torso registrations, and extracting measurements for all poses. Sample data and proposed processing are best visualized in a supplemental material video.

**Table 1:** Demographic details of study population

	Healthy controls		Scoliosis	
	Male	Female	Male	Female
*N*	9	11	12	14
Age (years)	14.6 (± 4.6)		14.7 (± 5.2)	
BMI	21.7 (± 7.6)		21.2 (± 9.2)	
Cobb angle	N/A		48.0 (± 40.46)	

### Surface reconstruction accuracy

On the optical breadboard, the RMS planar reconstruction error was 0.2 mm, while absolute landmark RMS error was 1.4 mm. The latter measure was influenced by difficulty in identifying the exact center of landmarks using the RGB texture map.

Rigid alignment between 3dMD reconstructions of the torso mannequin to the same phantom scanned in CT had an RMS error of 1.0 mm, approximately half the voxel size of the radiographic volume. These results, consistent with prior reports [37], demonstrate exceptional reconstruction accuracy for smooth surfaces.

### Rigid-body measurement accuracy

Topographic measurements performed on 3dMD reconstructions of the torso phantom were highly accurate compared to CT (Table 2). All intrinsic measurements were within 2% RMS error, apart from JN X-section area with 3.2% relative RMS error. BSR and principal axis were within 1° RMS error, while coronal centroid deviation was < 1 mm. Qangle had the worst accuracy overall with 3.2° RMS error. Note that these measurements only take into account reconstruction accuracy and stability of the measurement software, as torso registrations were performed with a standard nonrigid Iterative Closest Point algorithm and not the full registration algorithm, which requires a full-body scan.

**Table 2:** Phantom accuracy; two raters performed scans of a torso phantom in 3dMD with upright and prone positioning

		Mean	SD	CT	RMS Err
Intrinsic	Back area (dm^2^)	19.7	0.3	19.5	0.4
	Spine length (cm)	53.3	0.6	52.6	0.9
	XSA L2 (dm^2^) ♱	5.0	0.01	5.1	0.1
	XSA T8 (dm^2^) ♱	6.8	0.02	7.0	0.1
	XSA JN (dm^2^) ♱	5.2	0.07	5.4	0.2
	XSV L2-T8 (L) ♱	10.9	0.03	10.8	0.1
	XSV XP-JN (L) ♱	11.9	0.06	12.0	0.1
	XSV PSIS-JN (L) ♱	21.8	0.07	22.1	0.3
Pose-dependent	BSR 25 (°)	1.3	0.5	1.1	0.5
	BSR 50 (°)	0.2	0.8	0.3	0.8
	BSR 75 (°)	0.2	0.6	0.1	0.6
	BSR MAX (°)	1.5	0.3	1.5	0.3
	Centroid (mm) ♱	17.1	0.5	17.4	0.6
	Axis (°) ♱	4.0	0.1	4.5	0.5
	Qangle (°) ♱	7.9	2.2	10.3	3.2

Means and standard deviations (SD) are computed across both raters. The column labeled “CT” shows the ground truth measurement computed on a CT scan of the same phantom, while RMS Err shows the root-mean-squared error between topographic and CT measurements. Measurements marked ♱ are only performed in upright position for topographic scans

### Human subject reproducibility

Decoupling the variability contributed by subject posture vs the automated registration process is challenging, as the first may influence the second. To evaluate our registrations, we manually marked nine points on test–retest surface reconstructions and then inverted the atlas registration to map these points to the generic torso template (Table 3). The grand mean error was 5.4 mm, demonstrating consistent alignments at landmarked locations.

**Table 3:** Mean alignment error is measured by manually identifying nine fiducial landmarks (descriptions in text) on the surface reconstructions of repeated scans of the same subject (fiducial markers are left in place)

	EOS	A-pose	Adam
PSIS L	5.9	5.2	1.8
PSIS R	5.9	5.3	1.9
ASIS L	4.6	6.7	6.1
ASIS R	4.1	7.0	6.2
XP	6.7	7.1	1.2
JN	7.1	3.8	1.6
AC L	8.0	4.9	9.5
AC R	9.3	4.8	8.1
VP	4.9	7.3	2.9

These points are mapped into shared atlas space using the template torso registrations and the distance between repeated trials is reported in milimeters. Acromioclavicular joints (AC left and right) were landmarked by palpation but not used for topographic measurements

Surface topographic measurements were highly reliable with 80% of all ICC values ≥ 0.90 (Table 4). Descriptive statistics (grand mean and standard deviation) were tabulated across raters and trials for each parameter. Using a two-tailed t test, intra-rater ICCs for test–retest measurements were slightly higher than remove-replace (0.94 vs 0.92, *t* = 2.90, df = 71, *p* = 0.005 across both raters), but no difference was found between intra-rater remove-replace and inter-rater reliability (*t* = 1.08, df = 35, *p* = 0.28 for rater A; *t* = 0.86, df = 35, *p* = 0.40 for rater B).

**Table 4:** Reliability of surface-topographic measurements on human subjects. Results include both patients and controls

			Descriptives		ICC for intra-rater A		ICC for intra-rater B		ICC for inter-rater		
	Pose	Parameter	Grand mean	Grand SD	Test–retest	Remove–replace	Test–retest	Remove–replace	RaterA–RaterB	95% CI	
Intrinsic	EOS	Spine Length (cm)	47.9	3.8	1	0.99	1	0.98	0.98	0.96	0.99
		Back Area (dm^2^)	18.6	3.1	1	0.99	1	0.99	0.99	0.99	1
		XSA JN (dm^2^)	4.4	1.1	0.98	0.94	0.99	0.95	0.94	0.89	0.97
		XSA L2 (dm^2^)	5.9	1.2	1	1	1	1	1	0.99	1
		XSA T8 (dm^2^)	4.1	1.0	1	0.99	1	0.99	0.99	0.98	1
		XSV L2-T8 (L)	7.6	2.2	1	0.99	1	1	1	0.99	1
		XSV PSIS-JN (L)	9.2	2.3	1	1	1	0.99	0.99	0.99	1
		XSV XP-JN (L)	18.0	4.7	1	0.99	1	0.99	0.99	0.96	1
	A-pose	Spine Length (cm)	48.3	3.8	0.98	0.97	0.98	0.99	0.98	0.97	0.99
		Back Area (dm^2^)	16.6	2.7	0.99	0.99	0.99	0.99	0.99	0.98	1
		XSA JN (dm^2^)	4.4	1.2	0.98	0.97	0.97	0.98	0.98	0.96	0.99
		XSA L2 (dm^2^)	6.1	1.3	1	1	1	1	1	0.99	1
		XSA T8 (dm^2^)	4.1	0.9	0.99	1	1	0.99	0.99	0.97	0.99
		XSV L2-T8 (L)	7.8	2.1	0.99	1	1	0.99	1	0.99	1
		XSV PSIS-JN (L)	9.6	2.4	1	1	1	1	1	0.99	1
		XSV XP-JN (L)	17.6	4.6	0.99	1	1	0.99	0.99	0.98	0.99
	Adams bend	Spine Length (cm)	52.4	5.3	0.97	0.95	0.96	0.95	0.96	0.93	0.98
		Back Area (dm^2^)	22.2	3.8	0.99	0.98	0.99	0.99	0.99	0.98	0.99
Pose-dependent	EOS	BSR 25 (°)	− 2.9	8.2	0.97	0.97	0.98	0.92	0.94	0.9	0.97
		BSR 50 (°)	− 0.0	6.5	0.98	0.94	0.98	0.88	0.87	0.77	0.93
		BSR 75 (°)	− 3.7	4.9	0.92	0.8	0.9	0.49	0.65	0.44	0.79
		BSR MAX (°)	− 0.8	3.6	0.98	0.97	0.98	0.93	0.94	0.89	0.97
		Centroid (mm)	2.4	16.0	0.96	0.86	0.95	0.88	0.81	0.68	0.89
		Axis (°)	− 3.9	11.4	0.99	0.97	0.99	0.95	0.97	0.93	0.98
		Qangle (°)	− 3.2	15.3	0.73	0.63	0.88	0.81	0.61	0.38	0.77
	A-pose	BSR 25 (°)	− 0.6	8.0	0.95	0.95	0.96	0.94	0.95	0.91	0.97
		BSR 50 (°)	1.2	6.1	0.88	0.91	0.87	0.82	0.81	0.67	0.89
		BSR 75 (°)	− 2.4	4.8	0.66	0.61	0.66	0.71	0.57	0.34	0.74
		BSR MAX (°)	− 0.3	3.5	0.96	0.95	0.94	0.92	0.9	0.83	0.94
		Centroid (mm)	− 1.8	13.9	0.63	0.73	0.71	0.8	0.85	0.75	0.92
		Axis (°)	− 3.3	10.5	0.95	0.93	0.97	0.94	0.92	0.86	0.95
		Qangle (°)	5.3	15.4	0.67	0.78	0.66	0.7	0.49	0.22	0.68
	Adams bend	BSR 25 (°)	− 6.1	12.1	0.77	0.77	0.9	0.76	0.84	0.72	0.91
		BSR 50 (°)	1.1	7.6	0.97	0.97	0.98	0.96	0.95	0.91	0.97
		BSR 75 (°)	− 6.6	8.9	0.96	0.88	0.95	0.93	0.95	0.91	0.97
		BSR MAX (°)	− 4.9	6.1	0.95	0.81 ara>	0.94	0.96	0.95	0.9	0.97

Measurements are divided into intrinsic and pose-dependent classifications. Intrinsic measurements are isometrically invariant; that is, they are independent of changes in orientation and largely robust to minor changes in posture. Intraclass correlation coefficients (ICC) are computed within (intra-) and between (inter-) raters, along with 95% confidence intervals (CI). Grand means and standard deviations (SD) are computed across all scans and are presented in absolute units

Reliability for intrinsic measurements was nearly perfect, with an average inter-rater ICC of 0.99 across all poses and a minimum of 0.94 (X-section area at JN in EOS pose). Pose-dependent parameters were more variable, with Qangle in the A-pose having the lowest inter-rater ICC value (0.49). Reliability was not found to be dependent on body type; after applying Bonferroni–Holm correction for multiple comparisons, none of the surface-topographic parameters showed a significant correlation between consistency and BMI.^3^

Evaluating patients and controls separately, both groups averaged > 0.98 inter-rater ICC for intrinsic measurements. All other measures are expected to be zero for symmetric torso shapes, which explains why controls had lower ICCs than patients (0.58 vs 0.81 average inter-rater ICC).

### Case example

**Fig. 5:**
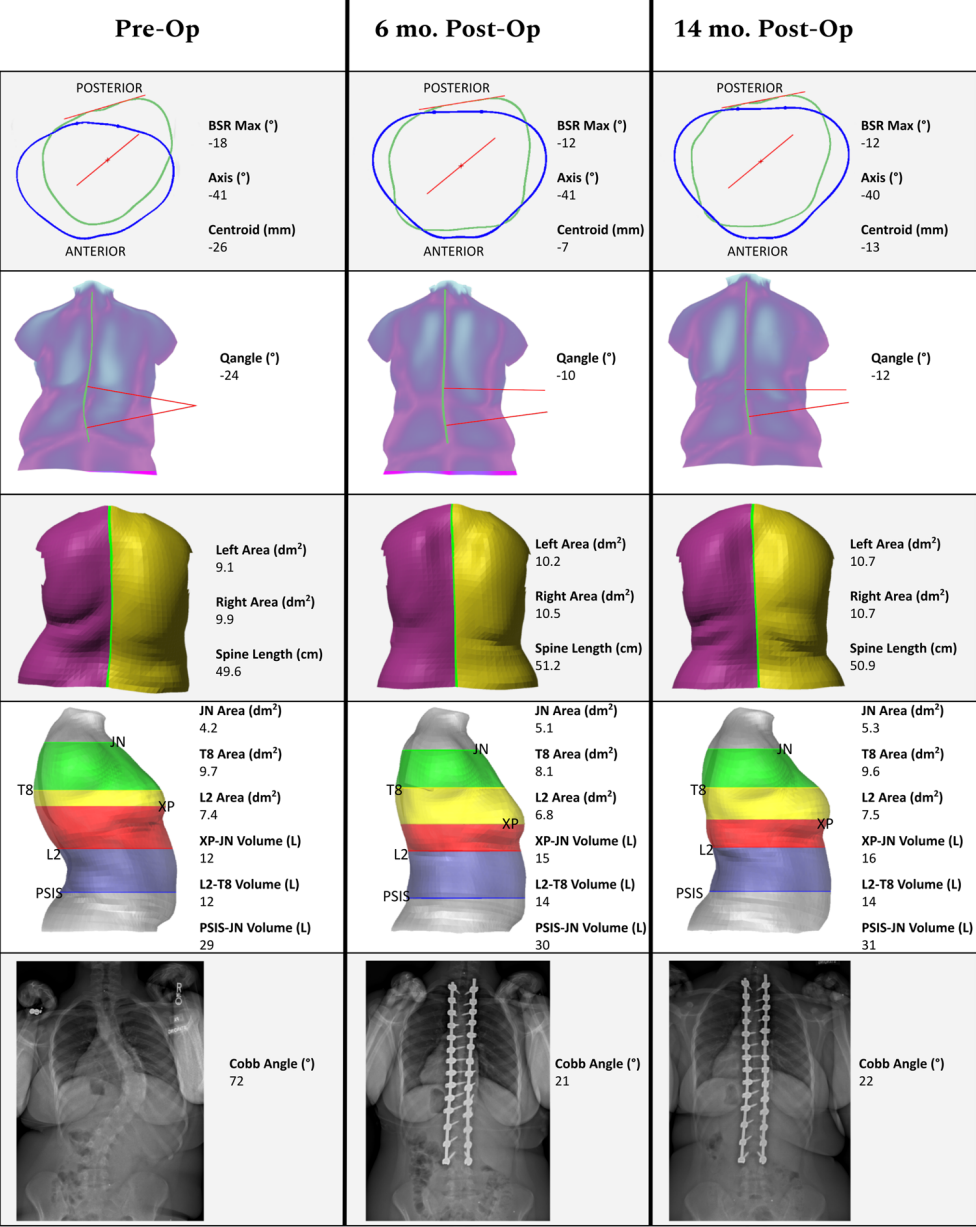
Case study topographic measurements, computed on A-pose scans. Rows from top to bottom: (1) axial measurements are computed at the level of maximal BSR (green outline) in the pre-op scan; outline at the level of PSIS (blue) shown for reference. (2) Qangle with asymmetry heatmap. (3) Back surface area and spine length. Note that post-operative changes in patient height may be reflected in reduced lordosis/kyphosis rather than spine length as computed on the surface. (4) Cross-sectional areas and section volumes, with landmarks used to choose axial cut levels shown. (5) Frontal radiographs from EOS scans collected on the same day as surface scans

The patient was a 15-year-old female with AIS who presented with a 72° right thoracic curve, ATR of 25°, 2 cm right shoulder elevation, waist-crease asymmetry, and a right thoracic prominence. She underwent PSF T2-L3 without surgical complications. Table 5 and Fig. 5 show how topographic measurements might be presented to a physician.

**Table 5:** Case study topographic measurements. In contrast to all other analysis in this study, BSR MAX for 6-month and 14-month follow-up scans is computed using the level of maximal BSR in the pre-op scan, defined as the fraction of the distance between PSIS and C7 landmarks

	Measurement	Pre-Op	6 Mo	14 Mo
EOS	Sp. Len. (cm)	52	52	51
	Back Area (dm^2^)	25	23	24
	XSA L2 (dm^2^)	7.9	6.8	8.0
	XSA T8 (dm^2^)	9.2	8.7	9.3
	XSA JN (dm^2^)	5.8	5.5	5.5
	XSV L2-T8 (L)	14	13	14
	XSV XP-JN (L)	14	15	14
	XSV PSIS-JN (L)	31	30	32
	BSR MAX (°)	− 17	− 10	− 9.0
	BSR 25 (°)	− 10	− 9	− 10
	BSR 50 (°)	− 13	− 8	− 10
	BSR 75 (°)	− 6	2	− 3
	Centroid (mm)	− 23	12	13
	Axis (°)	− 39	− 35	− 35
	Qangle (°)	− 21	− 12	− 9
A-pose	Sp. Len. (cm)	50	51	51
	Back Area (dm^2^)	19	21	21
	XSA L2 (dm^2^)	7.4	6.8	7.5
	XSA T8 (dm^2^)	9.7	8.1	9.6
	XSA JN (dm^2^)	4.2	5.1	5.3
	XSV L2-T8 (L)	12	14	14
	XSV XP-JN (L)	12	15	16
	XSV PSIS-JN (L)	29	30	31
	BSR MAX (°)	− 18	− 12	− 13
	BSR 25 (°)	− 13	− 9	− 9
	BSR 50 (°)	− 13	− 12	− 7
	BSR 75 (°)	− 7	− 7	− 2
	Centroid (mm)	− 26	− 7.4	13
	Axis (°)	− 41	− 41	− 40
	Qangle (°)	− 24	− 10	− 12
Adam	Sp. Len. (cm)	62	56	51
	Back Area (dm^2^)	31	27 ara>	28
	BSR MAX (°)	− 23	− 7	− 11
	BSR 25 (°)	− 4	− 7	− 5
	BSR 50 (°)	− 20	− 10	− 15
	BSR 75 (°)	− 18	− 2	− 6

## Discussion

This study establishes the reliability of a novel topographic scanner for assessment of scoliotic patients. An automated system producing rapid and reliable surface measurements has the potential to establish optical scanning as an important tool for objective measurement of body contour asymmetry in both clinical and research settings.

Optical scan time per subject was similar to biplane radiographs, and final measurements can be available even more quickly; radiographs were sent to EOS^®^ to generate a standard suite of validated radiographic measurements [38, 39]. Meanwhile, topographic scans can be processed on-site within minutes utilizing a markerless, fully automated, dedicated image processing workflow. The software takes as input surface data from any whole-body topographic scanner and similarly generates a reliable suite of surface measurements.

Experiments on rigid-body scan targets demonstrated the fidelity of the 3dMDbody scanner and robustness of the measurement tools. Fast capture speeds obviate motion artifacts, and reconstructions of smooth surfaces achieve sub-millimeter accuracy; intrinsic measurements performed on a phantom torso had an average RMS error of 1.7% compared with CT.

In our human trials, reliability was limited mostly by variations in posture; isometrically invariant parameters such as spine length and surface area demonstrated excellent reliability (ICC > 0.94) across all poses and raters. Reliability of other measurements was pose-dependent; average inter-rater ICC of BSR for A-pose was 0.81, but 0.92 for Adam’s bend pose. Careful choice of scan posture and simplification of patient instructions/positioning may further improve reliability of topographic measurements. It should be noted that standing radiographs suffer the same sensitivity to patient posture, and EOS scanners introduce additional variability from postural sway during a 5–20 s scan [40].

While other topographic scanners can operate in fully upright postures [24] or in full forward flexion [9], 3dMDbody can capture multiple positions without adjustment. Furthermore, scanning at 10 Hz may allow dynamic assessment of, e.g., bending, twisting, and inhalation/exhalation. The reliability of the system compares favorably with best-in-class topographic scanners: cross-sectional area and volume measurements can be compared directly to Table 3 from [26], with both systems achieving > 0.97 inter-rater ICCs on all comparable measurements.

In addition to further investigations of surface-based parameters, further studies are needed to assess relationships between surface-topographic measurements and spinal deformity patterns. Preliminary investigations (*N* = 105) show that, of the parameters discussed, BSR has the best correlation with Cobb angle with *R* = 0.72 (*p* < 10^–17^). This finding, in line with published work [24, 41], attests to the structural relationship between skeletal alignment and surface topography. However, the lack of strong linear correspondence also points to the complexity of this interaction; sophisticated modeling is required to make surface topography useful for clinical use like screening or detecting progression of scoliosis without radiation [9, 27].

However, even absent direct correspondence with radiographic parameters, we believe that the system presented here complements other imaging modalities by providing volumetric and surface-based measurements in load-bearing poses. Further development of volumetric analysis tools based on accurate and reliable 3D models may enable more objective appraisal of shoulder balance, waist-crease asymmetry, rib prominence, anterior chest wall asymmetry, and postural alignment. We believe that accurate 3D surface measurements will correlate more closely with body symmetry and patient self-image than standard radiographic measurements.

In conclusion, clinical surface parameters can be reliably measured in a fully automated system, paving the way for objective analysis of symmetry, body shape pre/post-surgery, and tracking of pathology without ionizing radiation.

## Supplementary Information

Below is the link to the electronic supplementary material.

Supplementary file1 (PDF 8166 kb)

Supplementary file2 (PDF 752 kb)

Supplementary file3 (MP4 62113 kb)

## Data Availability

The raw measurements used to compute reliability statistics are available upon request. The Spinal Alignment Registry is administered by an internal steering committee at HSS; researchers interested in collaborating in investigations using these data should contact Dr. Widmann.

## Footnotes

1 The full data-collection protocol included side bend and axial twisting poses, which will be addressed in future studies.

2 Any full-body surface scanner can be used; accuracy of topographic measurements will be limited by scanner speed and reconstruction accuracy. Our automated labeling and analysis software requires that surface geometry be represented as a triangulated mesh (e.g., STL, PLY, or OBJ files).

3 Spine length in EOS pose and T8 cross-sectional area in A-pose had raw *p* values of 0.05 and 0.02, respectively, which were adjusted to 1.58 and 0.89.

## Code availability

Code is not currently available for public dissemination. Interested parties should contact the corresponding author.

## References

[1] Haher TR, Gorup JM, Shin TM (1999). Results of the scoliosis research society instrument for evaluation of surgical outcome in adolescent idiopathic scoliosis. Spine.

[2] Sanders JO, Harrast JJ, Kuklo TR (2007). The spinal appearance questionnaire. Spine.

[3] Bago J, Sanchez-Raya J, Perez-Grueso FJSS (2010). The Trunk Appearance Perception Scale (TAPS): a new tool to evaluate subjective impression of trunk deformity in patients with idiopathic scoliosis. Scoliosis.

[4] King HA, Moe JH, Bradford DS (1983). The selection of fusion levels in thoracic idiopathic scoliosis. J Bone Joint Surg.

[5] Lenke L (2000). SRS terminology committee and working group on spinal classification revised glossary of terms.

[6] Lenke LG, Edwards CC, Bridwell KH (2003). The Lenke classification of adolescent idiopathic scoliosis: how it organizes curve patterns as a template to perform selective fusions of the spine. Spine.

[7] Jaremko JL, Poncet P, Ronsky J (2002). Genetic algorithm-neural network estimation of cobb angle from torso asymmetry in scoliosis. J Biomech Eng.

[8] Frerich JM, Hertzler K, Knott P (2012). Comparison of radiographic and surface topography measurements in adolescents with idiopathic scoliosis. Open Orthop J.

[9] Kokabu T, Kanai S, Kawakami N (2021). An algorithm for using deep learning convolutional neural networks with three dimensional depth sensor imaging in scoliosis detection. Spine J.

[10] Simony A, Hansen EJ, Christensen SB (2016). Incidence of cancer in adolescent idiopathic scoliosis patients treated 25 years previously. Eur Spine J.

[11] Illes T, Somoskeoy S (2012). The EOS(TM) imaging system and its uses in daily orthopaedic practice. Int Orthop.

[12] Luan FJ, Wan Y, Mak KC (2020). Cancer and mortality risks of patients with scoliosis from radiation exposure: a systematic review and meta-analysis. Eur Spine J.

[13] Sharma S, Andersen T, Wu C (2016). How well do radiologic assessments of truncal and shoulder balance correlate with cosmetic assessment indices in lenke 1C adolescent idiopathic scoliosis?. Clin Spine Surg.

[14] Mariconda M, Andolfi C, Cerbasi S (2016). Effect of surgical correction of adolescent idiopathic scoliosis on the quality of life: a prospective study with a minimum 5-year follow-up. Eur Spine J.

[15] Albay C, Kaygusuz MA (2021). Effect of instrumentation level on mental health subscale of scoliosis research society outcomes questionnaire in adolescent idiopathic scoliosis. Cureus.

[16] Nord F, Ferjencik R, Seifert B (2015). The 3dMD photogrammetric photo system in cranio-maxillofacial surgery: Validation of interexaminer variations and perceptions. J Cranio-Maxillofac Surg.

[17] Öwall L, Darvann TA, Larsen P (2016). Facial asymmetry in children with unicoronal synostosis who have undergone craniofacial reconstruction in infancy. Cleft Palate Craniofac J.

[18] Moyer HR, Carlson GW, Styblo TM (2008). Three-dimensional digital evaluation of breast symmetry after breast conservation therapy. J Am Coll Surg.

[19] Moreland MS, Pope MH, Wilder DG (1981). Moiré fringe topography of the human body. Med Instrument.

[20] Turner-Smith AR, Harris JD, Houghton GR (1988). A method for analysis of back shape in scoliosis. J Biomech.

[21] Fortin C, Feldman DE, Cheriet F (2010). Validity of a quantitative clinical measurement tool of trunk posture in idiopathic scoliosis. Spine.

[22] Poncet P, Delorme S, Ronsky JL (2001). Reconstruction of laser-scanned 3D torso topography and stereoradiographical spine and rib-cage geometry in scoliosis. Comput Methods Biomech Biomed Eng.

[23] Guidetti L, Bonavolontà V, Tito A (2013). Intra- and interday reliability of spine rasterstereography. Biomed Res Int.

[24] Knott P, Sturm P, Lonner B (2016). Multicenter comparison of 3D spinal measurements using surface topography with those from conventional radiography. Spine Deform.

[25] Tabard-Fougère A, Bonnefoy-Mazure A, Hanquinet S (2017). Validity and reliability of spine rasterstereography in patients with adolescent idiopathic scoliosis. Spine.

[26] Michalik R, Knod M, Siebers H (2020). Introduction and evaluation of a novel multi-camera surface topography system. Gait Posture.

[27] de Korvin G, Randriaminahisoa T, Cugy E (2014). Detection of progressive idiopathic scoliosis during growth using back surface topography: a prospective study of 100 patients. Ann Phys Rehabil Med.

[28] Bolzinger M, Bernardini I, Lemoine CT (2021). Monitoring adolescent idiopathic scoliosis by measuring ribs prominence using surface topography device. Spine Deform.

[29] Texas Department of State Health Services (2015). Spinal Screening Program (No. 1–111; Issue November)

[30] Rankine L (2012). Reproducibility of newly developed spinal topography measurements for scoliosis. Open Orthop J.

[31] Seoud L, Dansereau J, Labelle H (2013). Noninvasive clinical assessment of trunk deformities associated with scoliosis. IEEE J Biomed Health Inform.

[32] Horne JP, Flannery R, Usman S (2014). Adolescent idiopathic scoliosis: diagnosis and management. Am Fam Physician.

[33] Jaremko JL, Poncet P, Ronsky J (2002). Indices of torso asymmetry related to spinal deformity in scoliosis. Clin Biomech.

[34] Oxborrow NJ (2000). Assessing the child with scoliosis: the role of surface topography. Arch Dis Child.

[35] di Angelo L, di Stefano P, Vinciguerra MG (2011). Experimental validation of a new method for symmetry line detection. Comput-Aided Design Appl.

[36] Carreau JH, Bastrom T, Petcharaporn M (2014). Computer-generated, three-dimensional spine model from biplanar radiographs: a validity study in idiopathic scoliosis curves greater than 50 degrees. Spine Deform.

[37] Tzou CHJ, Artner NM, Pona I (2014). Comparison of three-dimensional surface-imaging systems. J Plast Reconstr Aesthet Surg.

[38] Melhem E, Assi A, El Rachkidi R (2016). EOS® biplanar X-ray imaging: concept, developments, benefits, and limitations. J Child Orthop.

[39] Kim SB, Heo YM, Hwang CM (2018). Reliability of the EOS imaging system for assessment of the spinal and pelvic alignment in the sagittal plane. Clin Orthop Surg.

[40] Blumer SL, Dinan D, Grissom LE (2014). Benefits and unexpected artifacts of biplanar digital slot-scanning imaging in children. Pediatr Radiol.

[41] Côté P, Kreitz BG, Cassidy JD (1998). A study of the diagnostic accuracy and reliability of the scoliometer and Adam’s forward bend test. Spine.

