# Supplementary material for: Reliability of automated topographic measurements for spine deformity
Source: Spine Deform. 2022 May 8;10(5):1035–45. doi: 10.1007/s43390-022-00505-9 (PMC9378338; doi:10.1007/s43390-022-00505-9)
Supplement: Supplementary file 1 — Supplementary file1 (PDF 8166 kb) [file 43390_2022_505_MOESM1_ESM.pdf]

# Appendix A: Overview of the 3dMD Full Body Scanner

## 1 Introduction

3dMD is a hardware/software company based in Atlanta, GA[1]. The product in question is the 3dMDbody system. Surface scans are collected using photogrammetry: 3D reconstructions from 2D images using the known geometry of the system. For full-body photogrammetric scanners, anywhere from dozens to hundreds of cameras may be employed; this model has 30. All cameras in the system are synchronized to fire simultaneously, controlled by a desktop computer. During scanning, images are captured at 10 frames per second; other versions of the 3dMD scanner are available that capture data at 60 or 120 fps.

## 2 Output

The 3dMD scanner produces two types of output data: raw images, and 3D surface reconstructions. Additional, subscription-based software is available from 3dMD to perform basic 3D measurements, but these ancillary capabilities are not discussed here.

### Raw Images

30 images per scan: 20 black and white images with a projected speckle pattern and 10 colored images. All 30 images for a single scan are captured within 1.7ms and are continuously captured at 10 frames per second. A single frame (30 image files) requires approximately 33Mb of storage space. See Figs 2a and 2b

### Surface Reconstructions

After raw data capture, frames can be selected for reconstruction; processing each frame takes approximately one minute. The result is a triangulated mesh. There will typically be about 250,00 vertices for a full-body scan, or around 3.8 faces/cm$^2$. NOTE: the meshes produced will NOT have the same topology from scan to scan. That is to say, you cannot directly compare vertex “n” between scans, even for the same subject, even in the same scan session. See Figs 1, 2c

## 3 Technical Specifications

1. **Physical Dimensions** 8x8x10 feet (height, width, length)

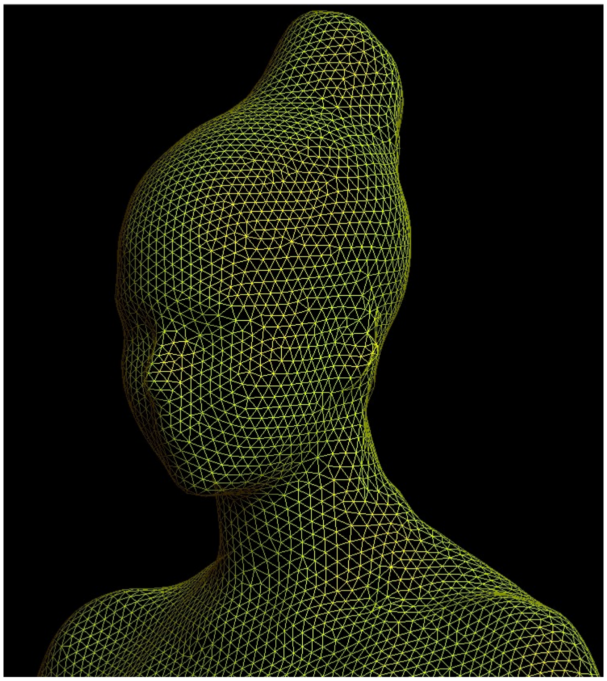

Figure 1: Wireframe mesh

2. **Surface Accuracy** Sub-millimeter. Validation studies have demonstrated 0.2mm surface accuracy, though the company only claims 0.7mm accuracy.
3. **Sampling Density** 250,000 vertices per surface reconstruction.
4. **Capture speed** 1.7ms for raw image capture.
5. **Sampling rate** 10 frames per second (3dMD also offers a different set of hardware, with similar specifications to this system in other ways but capable of 120hz capture rate).
6. **Scan Area** 2.2m x 1.2m x 2.2m (width x depth x height)
7. **File Types** PNG and JPG images for raw images, OBJ files for meshes (or STL, PLY, etc)
8. **File Sizes** 33Mb per frame for raw images, 50Mb per surface mesh
9. **Calibration Time** 90 seconds (Once per day)
10. **Reconstruction Time** About 1 min

[1] https://3dmd.com/products/body

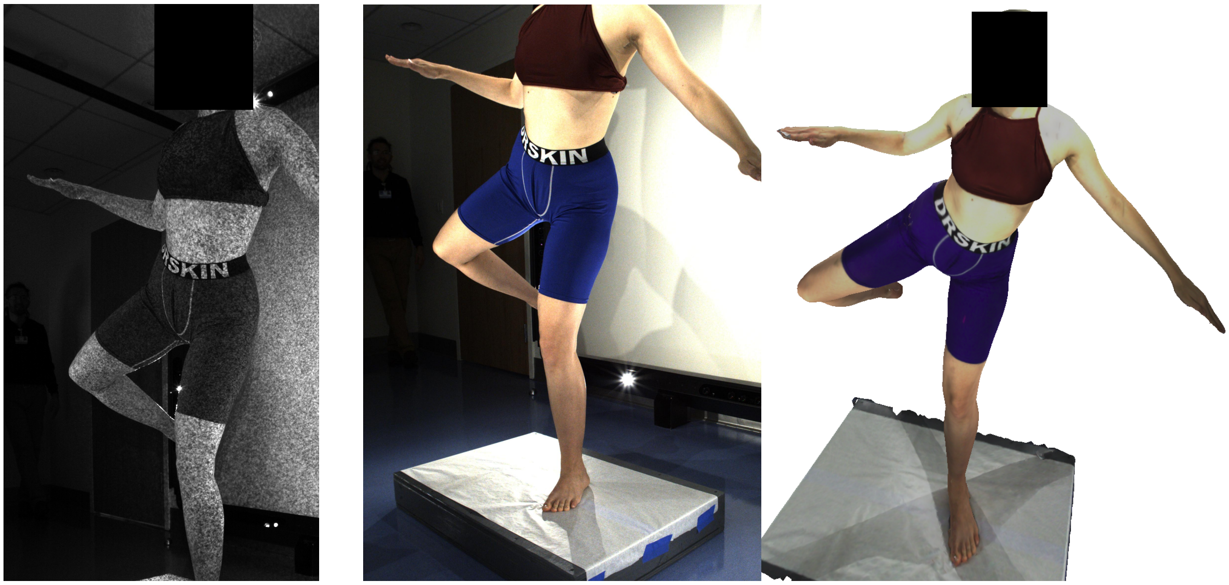

(a) Raw image with speckle pattern

(b) Raw image with color

(c) Reconstructed surface model with color texture

Figure 2: Sample 3dMD scan

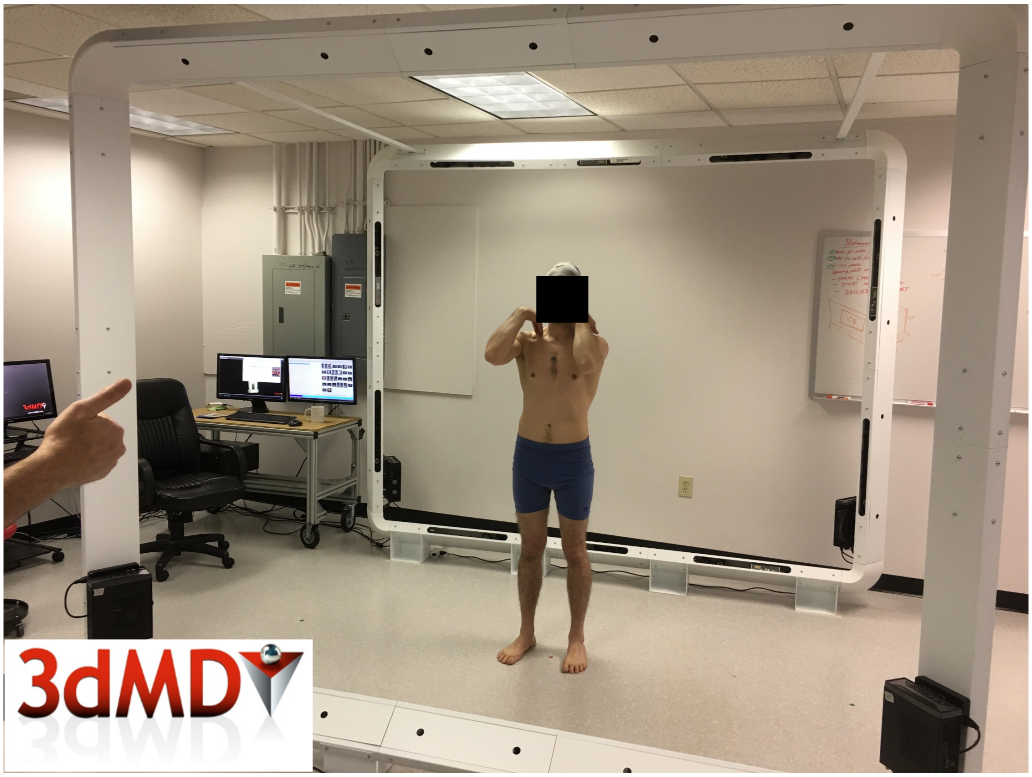


Figure 3: 3dMD full body surface scanner with workstation (left). Cameras are embedded in a steel frame for protection from accidental disturbance
