# Supplementary material for: Reliability of automated topographic measurements for spine deformity
Source: Spine Deform. 2022 May 8;10(5):1035–45. doi: 10.1007/s43390-022-00505-9 (PMC9378338; doi:10.1007/s43390-022-00505-9)
Supplement: Supplementary file 2 — Supplementary file2 (PDF 752 kb) [file 43390_2022_505_MOESM2_ESM.pdf]

Appendix B: Torso Correspondence from Articulated Body Model Registration

Our registration method is fully automated, employing pre-trained body models to rapidly predict correspondence between raw scans and a torso "atlas". This torso template is aligned and deformed to fit the scan data; subsequently, any desired measurements can be performed using pre-defined landmarks from the atlas.

Templates

For this study, we use the publicly available STAR model [1]. The template is defined as a triangulated mesh equipped with a pre-defined kinematic structure of 24 "joints". The surface mesh is articulated and rigged to those joints using modified animation techniques; full details of the model are provided in the original paper. For our purposes it is sufficient to understand that the model parameters are decomposed into an individual's body shape β and joint angles θ. By modifying the parameters β and θ we can generate realistic surface shapes in any desired posture. (Figure 1)

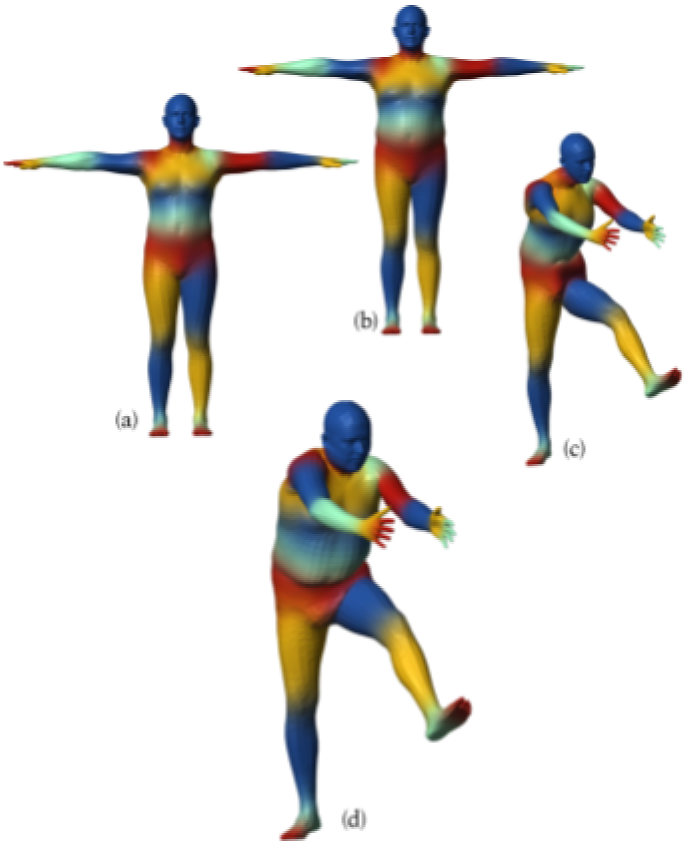

Figure 1. The STAR model. (a) the shared template surface (b) individual body shape β (c) template posed with joint angles **θ** (d) the final model is the composition of the template surface, the identity β, and the joint angles **θ**.

The published STAR model is augmented by mapping each point on the original template surface into a newly generated coordinate system in 4D. The mapping to 4D is computed with non-metric multidimensional scaling (MDS) to preserve geodesic distances

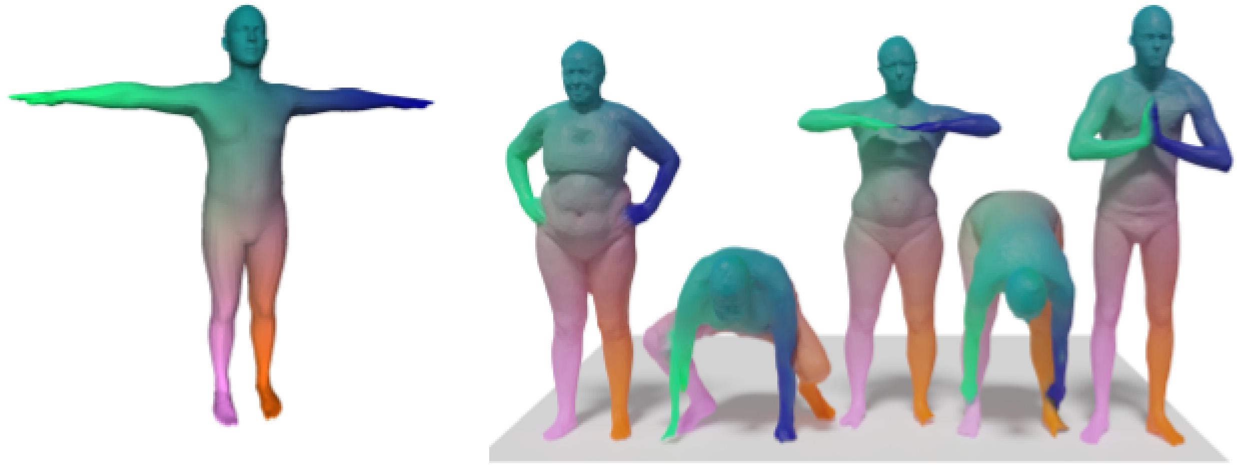

Figure 2. Left: template with coloration defined by MDS coordinates. Right: various scans from the FAUST dataset with automatic correspondence predictions.

on the template surface. The resulting “MDS space” is a euclidean space that approximates the original distances on the full-body template. (Figure 2)

Furthermore, we construct a torso template with known correspondence to the STAR template. (Figure 3) The torso model is topologically a cylinder, comprising 53 rings of 86 vertices each. The torso includes the shoulders and spans vertically from the top of the neck to the bottom of the pelvis. This model acts as an atlas of surface topography, because any number of anatomical landmarks can be labeled on the torso template; by registering this trunk model we automatically learn the locations of any points of interest.

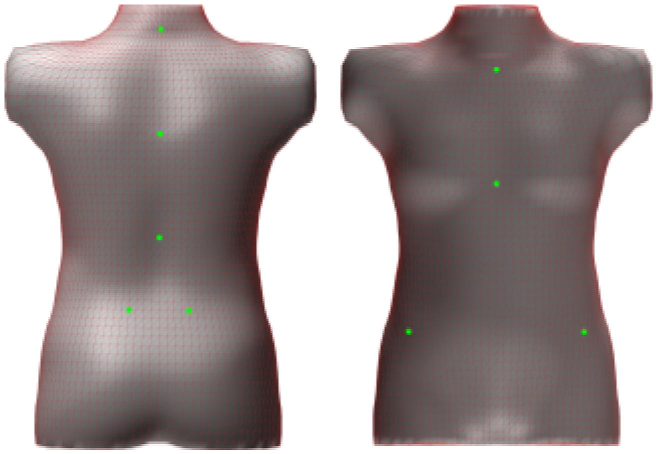

Figure 3. The template atlas is a regular triangulated mesh, structured as a regular grid. Landmarks are defined on the atlas, such that when the template is registered to a scan surface the landmark locations are automatically transferred.

Registration Algorithm

Algorithm Overview

- **Input:** A full-body surface scan of a human subject in an arbitrary pose. (Figure 4a) The data must be formulated as triangulated meshes, as is common for surface reconstructions.
- **Output:** A registered torso atlas fitted to match the 3D shape of the original scan. (Figure 4e)

1. Pre-processing: Scans are cleaned up, discarding loose patches and repairing non-manifold elements, then down-sampled. (Figure 4b)
2. Correspondence: Each point on the downsampled surface is mapped into template MDS space using a pre-trained deep mesh-convolutional network. (Figure 4c)
3. Model Optimization: STAR model parameters are optimized with a standard BFGS solver [2] to align the full-body template mesh with the scan data; correspondence matching uses the predicted MDS coordinates from step 2. (Figure 4d)
4. Torso Fitting: The trunk section of the full-body registered template is extracted and mapped to our torso atlas. This torso mesh is then fitted to the topography of the original scan data via standard nonrigid iterative closest point optimization. (Figure 4e)

Steps 1-3 in the above algorithm describe a general registration algorithm for full-body human correspondence matching. This registration method is described in detail in a pre-publication manuscript[1]. The final step, torso fitting, was tailored to the specific problem of automated clinical topographic measurements. Note that NO landmarks are used at any point in this registration process; the only input is the raw full-body surface scan.

[1] https://arxiv.org/abs/2108.06695

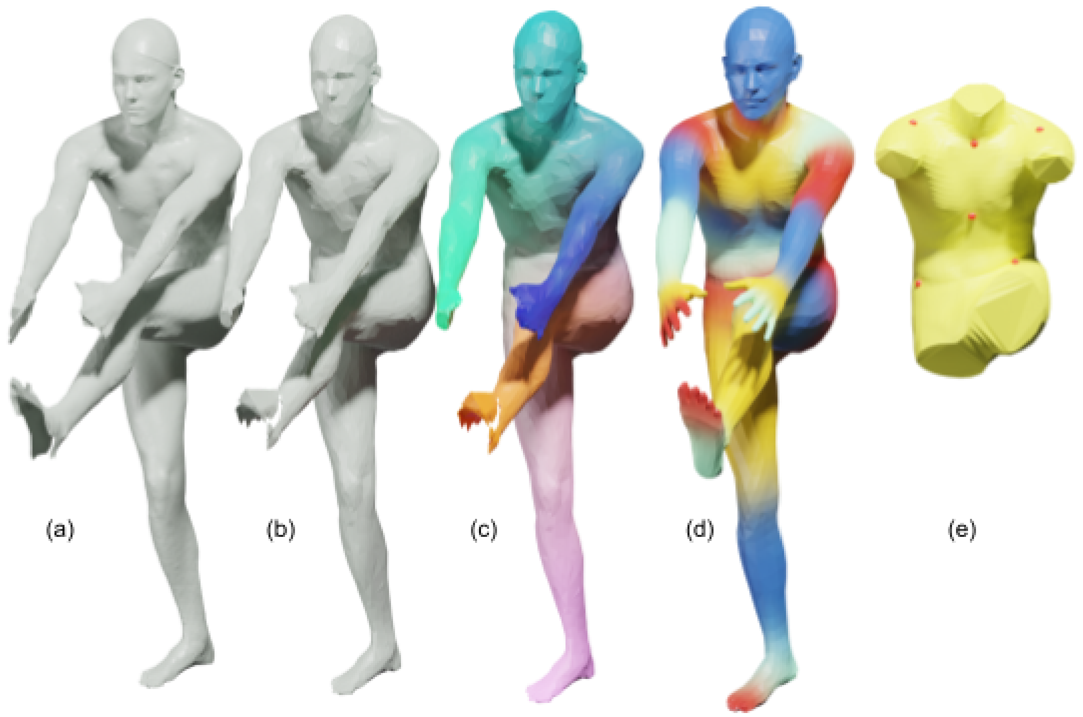

Figure 4. Registration pipeline. (a) Raw scan (b) pre-processed, downsampled scan with manifold topology (c) predicted correspondence from deep neural network (d) registered STAR model (e) extracted torso with automatically labelled landmark locations in red. The registration pipeline is fully automatic and can be run on any full-body mesh in arbitrary posture.

Measurement Software

A suite of measurement tools was developed in MATLAB to automatically extract topographic measurements from the registered torso atlas (Figure 3). Computing measurements on the registered atlas rather than the original scan surface has two primary advantages: (1) any point labeled on the atlas is automatically mapped to the registered surface (2) the atlas has a clean, fixed mesh topology that does not suffer from holes, discontinuities, or noise present in raw scans. Here we elaborate on the descriptions that are provided in the main paper.

I. **Spine Length** We measure the arclength on the surface of the torso running from the C7 marker to the midpoint of the PSIS markers. The arclength measurement follows the midline of the atlas, which is not necessarily aligned with the spinous processes.

II. **Back Area** Again, the region of interest is delimited by the area defined on the torso template. The dorsal region of the torso is bounded cranially by C7 and caudally by PSIS [3].

III. **Cross-Sectional Area** This measurement follows the definition from [4]. Transverse planes passing through the L2, T8, and JN markers are intersected with the torso surface. This results in three transverse slices, each containing the outline of the torso in the specified axial plane. The area contained in each outline is reported. In an effort to maintain consistency with prior work, we define the orientation of the axial plane with reference to the floor, though an anatomically based reference might allow this measurement to be expanded to other postures.

IV. **Section Volume** Once again we define this parameter following [4]. However, for brevity we do not compute all the sectional volumes described in that work. Each torso section is bounded above and below by axial slices as described for Cross-Sectional Area: (1) L2 to T8, (2) XP to JN, and (3) PSIS to JN.

V. **ATR/BSR Max** Trunk surface rotation is defined as the angle of a line lying tangent to the back surface [5, 6] compared to a reference plane. The reference plane chosen depends on the posture of the subject. For the Adam's forward bend posture, the plane of the floor is used as the reference, while upright postures use an anatomically-defined coronal plane as defined below. To find the maximal ATR, we compute the ATR at 100 levels between PSIS and C7 markers, discarding levels cranial to the acromia markers. We then report the maximal unsigned value.

VI. **ATR/BSR X%** Trunk surface rotation is measured (as above) at 100 levels. We report values at fixed levels: 25%, 50%, and 75% of the distance between PSIS and C7. At each level, we consider a range +/- 5% of the nominal value. For example, for the 25% value we find the maximum absolute value at 20-30% and report the signed value.

VII. **Centroid Deviation** Axial slices are found (as in Cross-Sectional Area) at 100 levels between PSIS and C7, again discarding levels above the acromia markers. At each slice, we find the lateral component of the centroid of the cross

section, similar to [7]. Values are computed with reference to the PSIS midpoint. We then report the maximum unsigned value of all slices considered.

VIII. **Trunk Axis** Cross sections are computed as in Centroid Deviation. We then find the principal axis of the cross section, similar to [7]. The angle of the principal axis is computed in reference to the subject's coronal plane as defined below. The maximal unsigned value is reported.

IX. **Qangle** This measurement was used in the Qantec [8] system as an analogue to Cobb angle; instead of measuring the curvature of the spine as defined by vertebral bodies, the Qangle uses the curve of the spinous processes on the back surface. To approximate the line of the spinous processes, we compute the back symmetry line as described in [9]. We then fit this curve with a 4th order harmonic function:

$$f(x) = c + a_0 sin(x/2) + b_0 cos(x/2) + a_1 sin(x) + b_1 cos(x) + a_2 sin(2x) + b_2 cos(2x) + a_3 sin(3x) + b_3 cos(3x)$$

This smoothed curve is then used to compute the Qangle between each pair of inflexion points; the maximal unsigned value is reported.

Anatomically-defined coronal plane: To define the coordinate axes, we use the following steps: (1) compute the normalized vector passing from left PSIS to right PSIS marker (2) compute the normalized vector passing from left ASIS to right ASIS marker (3) find the average of these vectors (4) The coronal plane is aligned to this averaged vector and is perpendicular to the floor.

References

[1] A.A.A. Osman, T. Bolkart, M.J. Black, STAR: Sparse Trained Articulated Human Body Regressor, (2020) 14–16. https://doi.org/10.1007/978-3-030-58539-6_36.

[2] M. Abadi, A. Agarwal, P. Barham, E. Brevdo, Z. Chen, C. Citro, G. Corrado, A. Davis, J. Dean, M. Devin, S. Ghemawat, I. Goodfellow, A. Harp, G. Irving, M. Isard, J. Yangqing, R. Jozefowicz, L. Kaiser, M. Kudlur, J. Levenberg, D. Mané, R. Monga, S. Moore, D. Murray, C. Olah, M. Schuster, J. Shlens, B. Steiner, I. Sutskever, K. Talwar, P. Tucker, V. Vanhoucke, V. Vasudevan, F. Viégas, O. Vinyals, P. Warden, M. Wattenberg, M. Wicke, Y. Yu, X. Zheng, {TensorFlow}: Large-Scale Machine Learning on Heterogeneous Systems, (2015). https://www.tensorflow.org/.

[3] L. Rankine, Reproducibility of Newly Developed Spinal Topography Measurements for Scoliosis, Open Orthop. J. 6 (2012) 226–230. https://doi.org/10.2174/1874325001206010226.

[4] R. Michalik, M. Knod, H. Siebers, M. Gatz, T. Dirrichs, J. Eschweiler, V. Quack, M. Betsch, Introduction and evaluation of a novel multi-camera surface topography system, Gait Posture. 80 (2020) 367–373. https://doi.org/10.1016/j.gaitpost.2020.06.016.

[5] L. Seoud, J. Dansereau, H. Labelle, F. Cheriet, Noninvasive clinical assessment of trunk deformities associated with scoliosis, IEEE J. Biomed. Heal. Informatics. 17 (2013) 392–401. https://doi.org/10.1109/TITB.2012.2222425.

[6] J.P. Horne, R. Flannery, S. Usman, Adolescent idiopathic scoliosis: Diagnosis and management, Am. Fam. Physician. 89 (2014) 193–198.

[7] J.L. Jaremko, Genetic Algorithm–Neural Network Estimation of Cobb Angle from Torso Asymmetry in Scoliosis, J. Biomech. Eng. 124 (2002) 496–503. https://doi.org/10.1115/1.1503375.

[8] N.J.N.J. Oxborrow, Assessing the child with scoliosis: The role of surface topography, Arch. Dis. Child. 83 (2000) 453–455. https://doi.org/10.1136/adc.83.5.453.

[9] L. di Angelo, P. di Stefano, M.G. Vinciguerra, Experimental validation of a new method for symmetry line detection, Comput. Aided. Des. Appl. 8 (2011) 71–86. https://doi.org/10.3722/cadaps.2011.71-86.
